# A prospective follow-up of thyroid volume and thyroiditis features on ultrasonography among survivors of predominantly mild to moderate COVID-19

**DOI:** 10.7717/peerj.15034

**Published:** 2023-03-17

**Authors:** Man Him Matrix Fung, David Tak Wai Lui, Keith Wan Hang Chiu, Sherman Haynam Lee, Chi Ho Lee, Wing Sun Chow, Alan Chun Hong Lee, Anthony Raymond Tam, Polly Pang, Tip Yin Ho, Carol Ho Yi Fong, Connie Hong Nin Loong, Chun Yiu Law, Kelvin Kai Wang To, Ching Wan Lam, Kathryn Choon Beng Tan, Yu Cho Woo, Ivan Fan Ngai Hung, Karen Siu Ling Lam, Brian Lang

**Affiliations:** 1 Department of Surgery, Queen Mary Hospital, University of Hong Kong, Hong Kong, China; 2 Department of Medicine, Queen Mary Hospital, University of Hong Kong, Hong Kong, China; 3 Department of Diagnostic Radiology, Queen Mary Hospital, University of Hong Kong, Hong Kong, China; 4 Department of Microbiology, Queen Mary Hospital, University of Hong Kong, Hong Kong, China; 5 Division of Chemical Pathology, Queen Mary Hospital, University of Hong Kong, Hong Kong, China; 6 Department of Pathology, Queen Mary Hospital, University of Hong Kong, Hong Kong, China

**Keywords:** COVID-19, SARS-CoV-2, Thyroid gland, Ultrasonography, Thyroiditis

## Abstract

**Background:**

We previously showed that higher SARS-CoV-2 viral load correlated with smaller thyroid volumes among COVID-19 survivors at 2 months after acute COVID-19. Our current follow-up study evaluated the evolution of thyroid volumes and thyroiditis features within the same group of patients 6 months later.

**Methods:**

Adult COVID-19 survivors who underwent thyroid ultrasonography 2 months after infection (USG1) were recruited for follow-up USG 6 months later (USG2). The primary outcome was the change in thyroid volume. We also reassessed thyroiditis features on USG, thyroid function and anti-thyroid antibodies.

**Results:**

Fifty-four patients were recruited (mean age 48.1 years; 63% men). The mean thyroid volume increased from USG1 to USG2 (11.9 ± 4.8 to 14.5 ± 6.2 mL, *p* < 0.001). Thirty-two patients (59.3%) had significant increase in thyroid volume by ≥15%, and they had a median increase of +33.3% (IQR: +20.0% to +45.0%). Multivariable logistic regression analysis showed that only higher baseline SARS-CoV-2 viral load independently correlated with significant thyroid volume increase on USG2 (*p* = 0.022). Among the seven patients with thyroiditis features on USG1, six (85.7%) had the features resolved on USG2. None had new thyroiditis features on USG2. All abnormal thyroid function during acute COVID-19 resolved upon USG1 and USG2.

**Conclusion:**

Most COVID-19 survivors had an increase in thyroid volume from early convalescent phase to later convalescent phase. This increase correlated with high initial SARS-CoV-2 viral load. Together with the resolution of thyroiditis features, these may suggest a transient direct atrophic effect of SARS-CoV-2 on the thyroid gland with subsequent recovery of thyroid volume and thyroiditis features.

## Introduction

Accumulating evidence from various cohort studies has demonstrated the spectrum of thyroid dysfunction in acute COVID-19 (Muller et al., 2020; Lania et al., 2020; Lui et al., 2021b), including reports of SARS-CoV-2-related thyroiditis. Mechanistically, this is supported by the expression of angiotensin-converting enzyme 2 (ACE2) mRNA in thyroid follicular cells, where ACE2 is the receptor for SARS-CoV-2 entry (Murugan & Alzahrani, 2021a; Murugan & Alzahrani, 2021b; Rotondi et al., 2021). Limited data available have suggested that most of the biochemical thyroid dysfunction during acute COVID-19 tends to improve after recovery (Pal et al., 2022).

In contrast to the impact of SARS-CoV-2 on the thyroid extensively evaluated from a biochemical perspective, less is known about the impact of SARS-CoV-2 on the thyroid from a structural perspective, especially among COVID-19 survivors with no biochemical thyroid dysfunction during acute COVID-19. This can be evaluated by using thyroid ultrasonography (USG). Such evaluation is clinically relevant, as ultrasonographic thyroiditis features could predict subsequent thyroid dysfunction (Rago et al., 2001) and would carry implications on the need for thyroid USG surveillance and even thyroid function monitoring among COVID-19 survivors.

We previously looked into the thyroid USG findings among 79 COVID-19 survivors at 2 months after acute COVID-19, the early convalescence phase (Lui et al., 2021a). We reported a 13.9% rate of thyroiditis features, and an independent correlation between higher SARS-CoV-2 viral load in acute COVID-19 and smaller thyroid volumes, suggesting a potential direct viral effect of SARS-CoV-2 on the thyroid. Recently, a similar study of 64 adult COVID-19 survivors evaluated with thyroid USG at median 5.7 months after acute COVID-19 revealed smaller thyroid volumes among COVID-19 survivors compared to healthy controls (Urhan et al., 2022). These raised concerns about the potential long-term sequelae of SARS-CoV-2 on the thyroid gland.

Following the results from the above two cross-sectional studies, it is crucial to clarify the implications of these observations in the later phase of convalescence (beyond 6 months after acute COVID-19) since thyroiditis features may precede thyroid dysfunction (Rago et al., 2001). To date, there is a paucity of long-term data on the evolution of thyroid USG findings among COVID-19 survivors, which is an important knowledge gap to fill as the scientific world is working on the evidence-based recommendations for the endocrine follow-up of COVID-19 survivors (Pal et al., 2022). As the population of COVID-19 survivors is growing rapidly, long COVID could evolve into a ‘pandemic of the pandemic’ (Murray, 2021). Results from such a follow-up study will inform the need for surveillance thyroid function and USG among COVID-19 survivors. Hence, we performed the current follow-up study of the same cohort of COVID-19 survivors from our previous study (Lui et al., 2021a) to evaluate the evolution of thyroid volumes and thyroiditis features on USG in the later phase of convalescence.

## Methods

This study followed the principles in the Declaration of Helsinki and was approved by the Institutional Review Board of the University of Hong Kong/Hospital Authority Hong Kong West Cluster (UW 21-213). All participants gave informed consent verbally before the commencement of the study.

### Recruitment of COVID-19 survivors

The recruitment of COVID-19 survivors for USG at 2 months after acute COVID-19 has been described in the previous publication (Lui et al., 2021a): 79 adult COVID-19 survivors (age ≥18 years), who attended the dedicated COVID-19 clinic of Queen Mary Hospital (a major COVID-19 centre) between 20 January 2021 and 7 April 2021, received their first thyroid USG (USG1) at around 2 months after acute COVID-19. The same group of patients were invited for a follow-up thyroid USG (USG2) around 6 months after USG1. It was not feasible to perform baseline thyroid USG during acute COVID-19 because all COVID-19 patients were treated in an isolation facility. Also, as patients with pre-existing thyroid diseases were excluded, the included patients would not have had a baseline thyroid USG before acute COVID-19.

### Thyroid USG

Both USG1 and USG2 were performed by the same trained operator, blinded to patients’ clinical information. When performing USG2, the same operator was unaware of the USG1 findings. The scanning and reporting protocol has been described in the previous publication (Lui et al., 2021a). The same ultrasound scanner (LOGIQ e, GE Healthcare, Wisconsin) equipped with a linear transducer (12L-SC; GE HealthCare, Chicago, IL, USA) was used. A standardized reporting protocol was adopted. Both grey-scale and colour Doppler images were interpreted in real-time. Dimensions of the thyroid lobes (width [W], depth [D] and length [L]) were measured and the volume of each thyroid lobe (V) was calculated by the formula V = W × D × L × 0.523 (Dighe et al., 2017). Total thyroid volume was calculated by the sum of the volume of both lobes. Isthmic volume was included in thyroid volume calculation if the isthmic anteroposterior diameter was >0.5 cm (Rago et al., 2018). Considering that the potential intra-observer variability of thyroid volume measurement by USG could vary from ±14% to from −9% to +16.4% in the literature (Schlögl et al., 2006; Andermann et al., 2007), in this study, significant thyroid volume change was defined as ±15% change to accommodate for the maximal intra-observer error. Normal appearance of the thyroid on USG was defined as a homogeneous parenchyma hyperechoic to the surrounding strap muscles. Thyroiditis features include diffuse reduction or diffuse heterogeneous changes in thyroid parenchymal echogenicity (Shin, Kim & Lee, 2010), diffuse micro-nodular changes (Yeh, Futterweit & Gilbert, 1996) and diffuse vascularity changes (Rago et al., 2018; Alexander et al., 2020). The echogenicity of the strap muscles was used as reference to describe thyroid echogenicity. Diffuse micro-nodular changes were defined as micro-nodules (1–6 mm) diffusely distributed throughout the thyroid parenchyma. Vascularity was assessed with colour Doppler, with the frequency and gain adjusted to a level with the highest sensitivity and yet the least random noise (Rago et al., 2018). Vascularity was graded as reduced, normal, moderately increased, clearly increased and thyroid inferno as previously described (Rago et al., 2018). Abnormal vascularity was defined as increased or reduced vascularity patterns. Ultrasonographic features of individual macro-nodules (nodules with the shortest dimension ≥1 cm) were not considered thyroiditis features. Thyroid nodules, if present, were evaluated according to the 2015 American Thyroid Association guidelines (Haugen et al., 2016), in which the decision of fine needle aspiration was based on the level of suspicion on sonographic pattern and the size of the nodules.

### Diagnosis of COVID-19 and SARS-CoV-2 viral load measurement

This has been described in the previous publication (Lui et al., 2021a). All patients had the presence of SARS-CoV-2 confirmed by reverse transcription-polymerase chain reaction (RT-PCR) from the nasopharyngeal swab (NPS) or deep throat saliva (DTS), using the LightMix SarbecoV E-gene assay (TIB Molbiol, Berlin, Germany), which targeted the envelope protein (E) gene of SARS-CoV-2 as previously described. Cycle threshold (Ct) values were obtained from the qualitative LightMix SarbecoV E-gene assay (TIB Molbiol, Berlin, Germany) performed on specimens from NPS or DTS (whichever was lower). The Ct value represents the number of cycles required for a gene target or a PCR product to be detected. While viral loads were not directly measured with a dedicated quantitative RT-PCR assay in this analysis, studies have shown a good correlation between Ct values and SARS-CoV-2 viral loads (Lui et al., 2021c; Lui et al., 2021d), such that the lower the Ct values, the higher the viral loads. COVID-19 severity was classified according to the ‘Chinese Clinical Guidance for COVID-19 Pneumonia Diagnosis and Treatment (7th edition)’ published by the Chinese National Health Commission (Lui et al., 2021b).

### Assessment of thyroid function and autoimmunity

This has been described in the previous publication (Lui et al., 2021a). Serum TSH, fT4 and fT3 were measured with immunoassays ADVIA Centaur^®^ TSH3-Ultra, FT4 and FT3 assays, respectively (Siemens Healthcare Diagnostics Inc., Erlangen, Germany). The reference ranges for TSH, fT4 and fT3 were 0.35–4.8 mIU/L, 12–23 pmol/L and 3.2–6.5 pmol/L, respectively. Non-thyroidal illness syndrome (NTIS) was defined by low fT3 with normal/low TSH. Anti-thyroglobulin (anti-Tg) and anti-thyroid peroxidase (anti-TPO) antibody titres were measured with QUANTA Lite^®^ Thyroid T and TPO enzyme-linked immunosorbent assay, respectively (Inova Diagnostics, San Diego, CA, USA). Positive anti-Tg and anti-TPO was defined by >100 units.

### Definitions of comorbidities

This has been described in the previous publication (Lui et al., 2021a). Both active smokers and ex-smokers were considered to have smoking habits. Both chronic drinkers and ex-drinkers were considered to have drinking habits. Body weight and height were measured at the time of thyroid USG. Obesity was defined by an Asian-specific body mass index (BMI) cut-off of 27.5 kg/m^2^. Diabetes was defined by a known diagnosis of diabetes or HbA1c ≥6.5% on admission. Charlson comorbidity index was calculated for each patient (Yang et al., 2016).

### Study outcomes

The primary outcome was the change in thyroid volume from USG1 to USG2.

Secondary outcomes included:

(i) Factors associated with a significant thyroid volume change from USG1 to USG2 (defined as a ±15% change);
(ii) The evolution of thyroiditis features from USG1 to USG2; and
(iii) Factors associated with the persistence of thyroiditis features at USG2.

### Statistical analyses

All statistical analyses were performed with IBM^®^ SPSS^®^ version 20. Two-sided *p*-values <0.05 were considered statistically significant. Data were presented as mean with standard deviation (SD), median with interquartile range (IQR), or number with percentage as appropriate. Between-group comparisons were performed with paired *t*-test for continuous variables as appropriate, and Chi-square, Fisher’s exact or McNemar tests for categorical variables as appropriate. Data not normally distributed were transformed logarithmically before analyses. Multivariable logistic regression analysis was used to identify the variables independently associated with significant thyroid volume increase. All variables with statistical significance in the univariate analysis (*p* < 0.05) were included in the multivariable regression analysis.

### Patient and public involvement

Patients received USG assessments of thyroid gland at 2 months after acute COVID-19 (USG1), and 6 months after USG1 (USG2). They were recruited from the COVID-19 clinic of Queen Mary Hospital and participated in the study on a voluntary basis. The research outcome measures were established based on our previous study (Lui et al., 2021a).

## Results

### Baseline characteristics

Fifty-four (68.4%) out of the 79 patients from our previous study (Lui et al., 2021a) turned up for USG2 and were included in the current analysis. The remaining 25 patients (31.6%) from the first study refused to attend USG2 and were excluded. The included 54 patients and the excluded 25 patients did not differ in terms of age (*p* = 0.131), sex preponderance (*p* = 0.461), BMI (*p* = 0.125), initial TSH (*p* = 0.414), fT4 (*p* = 0.768), CRP levels (*p* = 0.071), SARS-CoV-2 viral loads (*p* = 0.159) and the clinical severity of acute COVID-19 (*p* = 0.470).

The baseline characteristics of the included 54 individuals are summarized in Table 1. None had been vaccinated against SARS-CoV-2. All were hospitalized during acute COVID-19. On admission, 32 (59.3%) had mild disease, 21 (38.9%) had moderate disease and 1 (1.9%) had severe disease clinically. During hospitalization, only two (3.7%) subsequently required intensive care unit admission. Twenty-one (38.9%) patients received remdesivir, 20 (37.0%) received interferon beta-1b and 10 (18.5%) received dexamethasone to treat COVID-19. At the time of acute COVID-19, 6 patients (17.1%; out of 35 valid thyroid function tests) had abnormal thyroid function on admission: 2 had mildly low TSH (TSH 0.11 mIU/L, fT4 15 pmol/L, fT3 3.3 pmol/L; and TSH 0.24 mIU/L, fT4 13 pmol/L, fT3 3.2 pmol/L) and 4 had isolated low fT3 typical of NTIS. None had overt thyrotoxicosis or hypothyroidism. None had anterior neck pain typical of subacute thyroiditis. Notably, all thyroid function abnormalities resolved by the time of USG1. None had re-infection, re-hospitalization, or vaccination against SARS-CoV-2 by the time of USG2.

**Table 1:** Baseline clinical characteristics of the COVID-19 survivors who received both thyroid ultrasonography at 2 months and 9 months after COVID-19 (*n* = 54).

Age (years)	48.1 ± 14.7
Male	34 (63.0%)
Smoking	6 (11.1%)
Drinking	10 (18.5%)
Charlson comorbidity index	
0	46 (85.2%)
≥1	8 (14.8%)
Hypertension	10 (18.5%)
Diabetes	4 (7.4%)
Initial admission for COVID-19	
Symptomatic	44 (81.5%)
Baseline clinical severity	
Mild	32 (59.3%)
Moderate	21 (38.9%)
Severe	1 (1.9%)
Baseline SARS-CoV-2 PCR Ct value	23.45 (17.60–28.60)
TSH (mIU/L)^a^	0.87 (0.61–1.50)
fT4 (pmol/L)^a^	17.0 (15.0–19.0)
fT3 (pmol/L)^a^	3.9 (3.4–4.2)
Abnormal TFTs	6/35 (17.1%)
Anti-TPO positivity	4/34 (11.8%)
Anti-Tg positivity	4/34 (11.8%)
Elevated CRP	20 (37%)
Length of stay (days)	9 (6–13)
Exposure to COVID-19 treatments	
Remdesivir	21 (38.9%)
Interferon beta-1b	20 (37.0%)
Dexamethasone	10 (18.5%)
Ribavirin	7 (10.1%)
Clofazimine	4 (5.1%)
Tocilizumab	1 (1.3%)
Intensive care unit admission	2 (3.7%)

**Notes.**

Data are presented as mean ± standard deviation, median (interquartile range) and number (percentage) as appropriate.

Abbreviations Ct cycle threshold TSH thyroid-stimulating hormone fT4 free thyroxine fT3 free triiodothyronine TFTs thyroid function tests TPO thyroid peroxidase Tg thyroglobulin CRP C-reactive protein

a *n* = 35.

Table 2 shows the changes in sonographic findings and laboratory parameters from USG1 to USG2. USG1 and USG2 were performed at a median of 2.4 and 8.7 months after acute COVID-19 respectively. Serum TSH increased from acute COVID-19 to USG1 (*p* < 0.001) and USG2 (*p* < 0.001). Similar trend was observed for fT3 from acute COVID-19 to USG1 (*p* < 0.001). All abnormal thyroid function during acute COVID-19 resolved upon USG1 and USG2. Elevated CRP at acute COVID-19 largely resolved at USG1 (only one had elevated CRP), and completely resolved at USG2. In this cohort, none of the thyroid nodules required fine needle aspiration according to the 2015 American Thyroid Association guidelines.

**Table 2:** Comparing thyroid USG findings and blood results between acute COVID-19 and USG1/USG2 (*n* = 54).

	**Acute COVID-19**	**USG1**	**USG2**	***P* value (USG1 vs USG2)**
Interval from acute COVID-19 (months)	–	2.4 (1.6–3.6)	8.7 (8.0–9.3)	–
Thyroid volume (mL)	–	11.9 ± 4.8	14.5 ± 6.2	**<0.001**
Thyroiditis features	–	7 (13.0%)	1 (1.9%)	**0.031**
BMI (kg/m^2^)	–	26.3 (22.8–29.6)	25.4 (21.9–30.3)	0.070
TSH (mIU/L)^a^	0.87 (0.61–1.50)^b^	1.45 (0.91–1.90)^c*^	1.51 (0.96–1.90)^d*^	0.353
fT4 (pmol/L)	17.0 (15.0–19.0)^b^	17.0 (15.5–18.5)^c^	18.0 (16.0–19.0)^d^	0.267
fT3 (pmol/L)	3.9 (3.4–4.2)^b^	4.9 (4.6–5.2)^c*^	–	–
Abnormal TFTs	6/35 (17.1%)	0/45 (0%)	0/54 (0%)	–
Anti-TPO positivity	4/34 (11.8%)	5/37 (13.5%)	5/37 (13.5%)	>0.999
Anti-Tg positivity	4/34 (11.8%)	2/37 (5.4%)	1/37 (2.7%)	>0.999
Elevated CRP	20 (37.0%)	1 (1.9%)^*^	0 (0%)	>0.999

**Notes.**

a Logarithmically transformed before analysis.

b *n* = 35.

c *n* = 45.

d *n* = 54.

* *p* < 0.001 compared with acute COVID-19.

Abbreviations BMI body mass index TFTs thyroid function tests CRP C-reactive protein

Continuous variables were compared with paired *t*-tests; categorical variables compared with McNemar’s test.

### Changes in thyroid volume over time

**Figure 1:**
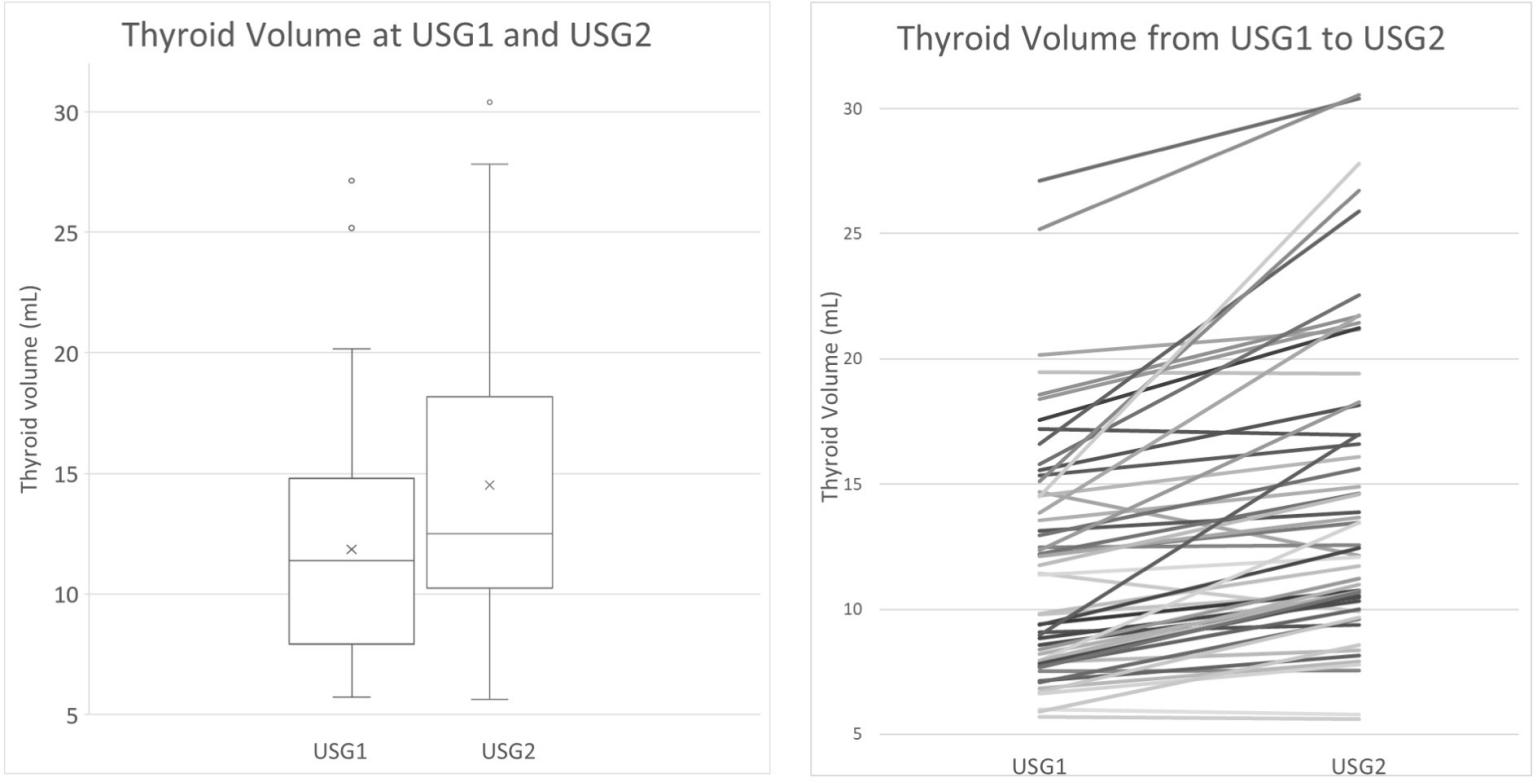
Distribution and evolution of thyroid volumes at USG1 and USG2. Left panel: the distribution of thyroid volumes at USG1 and USG2; Right panel: the evolution of thyroid volume of each individual from USG1 to USG2.

**Figure 2:**
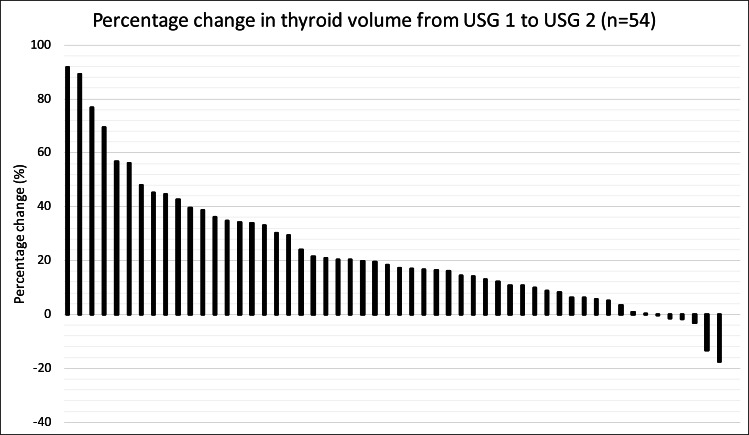
Waterfall plot illustrating the changes in thyroid volume among the 54 included COVID-19 survivors from USG1 to USG2. Thirty-two patients (59.3%) had a significant increase (≥15%) in thyroid volume (median +33.3%; IQR +20.0% to +45.0%). Only 1 patient (1.9%) had a significant reduction in thyroid volume (−17%).

**Figure 3:**
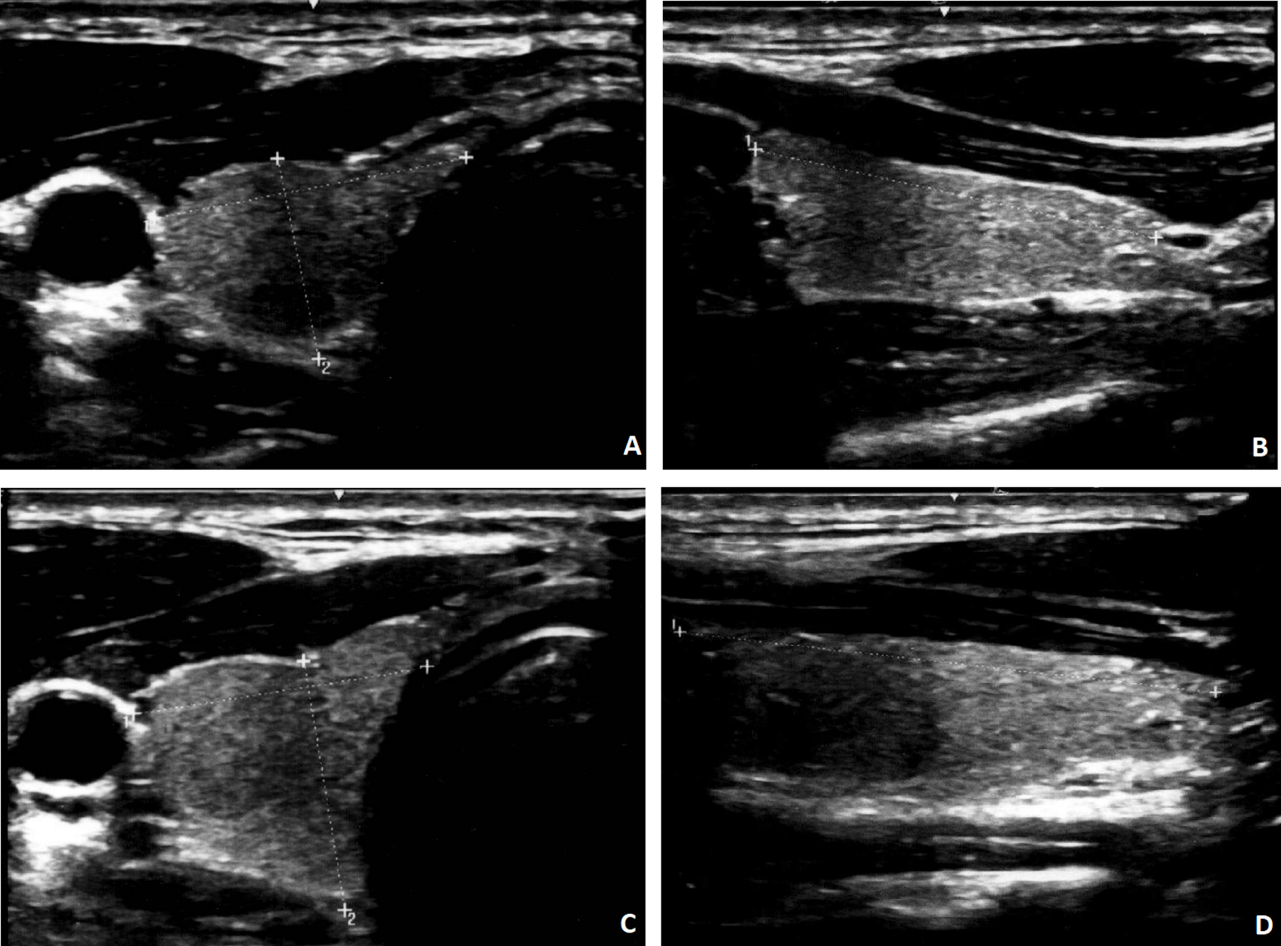
Comparison of USG1 and USG2 of a COVID-19 survivor who had interval enlargement of right thyroid volume by 48%. (A) Transverse and (B) longitudinal views of the right thyroid lobe at USG1, with the right lobe measuring 1.72 × 1.19 × 3.56 cm (volume = 3.81 mL); (C) transverse and (D) longitudinal views of the right thyroid lobe at USG2, with the right lobe measuring 1.84 × 1.46 × 4.00 cm (volume = 5.62 mL).

The mean thyroid volume of the cohort increased from USG1 to USG2 (from 11.9 ± 4.8 to 14.5 ± 6.2mL, *p* < 0.001) (Table 2; Fig. 1). The evolution of thyroid volume from USG1 to USG2 is illustrated in Fig. 1. Thirty-two patients (59.3%) had a significant increase in thyroid volume (defined by ≥15% increase), while only 1 (1.9%) had a significant reduction in thyroid volume (−17%) (Fig. 2). Among the 32 patients with significant increase in thyroid volume, the median percentage increase of thyroid volume from USG1 to USG2 was +33.3% (IQR +20.0% to +45.0%). Despite the overall increase in thyroid volume over time, none of the patients experienced compressive symptoms. The serial images of the right thyroid lobe of one of the COVID-19 survivors who had significant thyroid volume increase from USG1 to USG2 was illustrated in Fig. 3. Comparison between mild and non-mild COVID-19 cases revealed no significant differences in the thyroid volume changes (mild: 24.5% ±20.7 *vs* non-mild: 22.7% ±25.7, *P* = 0.653) nor thyroid volumes at USG2 (mild: 14.2ml ±6.5 *vs* non-mild: 14.8ml ±6.05, *p* = 0.573). Comparison of patients who did and did not have significant increase in thyroid volume (Table 3) showed that patients with significant increase in thyroid volume had higher SARS-CoV-2 viral load in acute COVID-19 (*p* = 0.004), lower CRP in acute COVID-19 (*p* = 0.014) and less dexamethasone use (*p* = 0.010). Baseline thyroid function and autoimmunity did not differ. Multivariable logistic regression analysis (Table 4) showed that only higher SARS-CoV-2 viral loads in acute COVID-19 remained to be the independent factor associated with a significant increase in thyroid volume (*p* = 0.022). Exclusion of the COVID-19 survivor with a significant reduction in thyroid volume resulted in consistent conclusions (Tables S1 and S2).

**Table 3:** Comparison between patients with and without significant thyroid volume increase.

	**Significant increase in thyroid volume**	**No significant increase in thyroid volume**	***P* value**
Number of patients	32	22	–
Age (year)	48.7 ± 16.1	47.3 ± 12.6	0.746
Male	20 (62.5%)	14 (63.6%)	0.932
BMI (kg/m^2^)^a^	25.7 (22.6–29.1)	28.2 (23.5–30.1)	0.411
Charlson Comorbidity index ≥1	5 (15.6%)	3 (13.6%)	>0.999
Hypertension	5 (15.6%)	5 (22.7%)	0.509
Diabetes mellitus	1 (3.1%)	1 (4.5%)	>0.999
Smoking	5 (15.6%)	1 (4.5%)	0.383
Drinking	6 (18.8%)	4 (18.2%)	>0.999
Parameters in acute COVID-19			
SARS-CoV-2 PCR Ct value^a^	19.9 (15.6–28.3)	26.9 (21.0–29.1)	**0.004**
CRP (mg/dL)^a^	1.02 (0.32–2.82)	2.21 (0.55–6.70)	**0.014**
TSH (mIU/L)^a^	0.87 (0.73–1.50)^b^	0.85 (0.55–1.33)^c^	0.592
fT4 (pmol/L)	17.0 (15.0–18.5)^b^	17.0 (15.0–19.0)^c^	0.896
fT3 (pmol/L)	3.8 (3.4–4.4)^b^	4.1 (3.8–4.3)^c^	0.543
Anti-TPO positivity	3/21 (14.3%)	1/13 (7.7%)	0.502
Anti-Tg positivity	3/21 (14.3%)	0/13 (0%)	0.144
Baseline COVID-19 severity			0.087
Mild	22 (68.8%)	10 (45.5%)	
Non-mild	10 (31.2%)	12 (54.5%)	
COVID-19 treatments			
Remdesivir	11 (34.4%)	10 (45.5%)	0.571
Interferon beta-1b	11 (34.4%)	9 (40.9%)	0.514
Dexamethasone	2 (6.3%)	8 (36.4%)	**0.010**
Ribavirin	3 (9.4%)	4 (18.2%)	0.425
Clofazimine	2 (6.3%)	2 (9.1%)	0.541
Tocilizumab	0 (0%)	1 (4.5%)	0.407

**Notes.**

a Logarithmically transformed before analysis.

b *n* = 14.

c *n* = 21.

Abbreviations BMI body mass index CRP C-reactive protein

Note: *P*-values set in boldface indicate statistical significance.

**Table 4:** Multivariable logistic regression analysis of the factors in COVID-19 associated with subsequent significant increase in thyroid volume.

	**Adjusted odds ratio** **(95% CI)**	***P* value**
SARS-CoV-2 viral load in acute COVID-19^a,b^	17.6 (1.50–207)	**0.022**
Baseline CRP in acute COVID-19 (mg/dL)^a^	0.78 (0.42–1.44)	0.425
Dexamethasone treatment in acute COVID-19	0.18 (0.02–1.37)	0.097

**Notes.**

*p* = 0.116 in Hosmer and Lemeshow test.

a Logarithmically transformed before analysis.

b SARS-CoV-2 viral loads were presented as $ \frac{1}{\text{SARS-CoV-2 PCR Ct value}} $.

Abbreviation CRP C-reactive protein

Note: *P*-values set in boldface indicate statistical significance.

### Evolution of thyroiditis features

Seven patients (13.0%) had thyroiditis features at USG1, with their clinical profiles summarized in Table 5. All patients with thyroiditis features on USG1 had completely resolved on USG2, except one (patient 1, Table 5). Interestingly, this was the only patient with positive anti-Tg. Hence, the thyroiditis features could be related to the underlying thyroid autoimmunity. Comparison between patients with and without persistent thyroiditis features revealed the only difference being numerically lower fT3 levels in acute COVID-19 among those with persistent thyroiditis features (with persistent thyroiditis features: 2.5 pmol/L *vs* without persistent thyroiditis features: 4.0 pmol/L), although the statistical significance of this comparison may be limited by the small sample size.

**Table 5:** Clinical profiles of COVID-19 survivors with thyroiditis features on USG1.

**Acute COVID-19**												**Thyroiditis features on USG1**	**Results at the time of USG2**					
	**Sex/ Age**	**BMI**	**CRP**	**TSH**	**fT4**	**fT3**	**Anti-TPO**	**Anti-Tg**	**Rx**	**SARS-CoV-2 PCR Ct value**	**Baseline COVID-19 Severity**		**Thyroiditis features**	**TSH**	**fT4**	**Anti-TPO**	**Anti-Tg**	**TV Change**
1	F/54	18.8	**2.88**	1.5	13	**2.5**	Neg	**Pos**	I + RM	20.4	Moderate	E + M	Persisted	1.6	17	Neg	**Pos**	+39.6%
2	F/52	20.0	**6.11**	0.87	23	4.1	Neg	Neg	Nil	29.1	Moderate	E	Resolved	0.73	18	Neg	Neg	+45.2%
3	F/48	31.6	0.74	0.77	18	4.3	N/A		RM	35.6	Moderate	E + M	Resolved	1.4	17	Neg	Neg	+10.7%
4	M/47	25.2	**2.82**	2.30	14	3.8	Neg	Neg	I + RM	23.5	Moderate	V^a^	Resolved	2.4	20	Neg	Neg	+5.2%
5	M/57	25.7	0.34	N/A					Nil	13.4	Mild	E	Resolved	1.2	15	N/A		+18.5%
6	M/64	24.2	**5.50**	0.71	17	3.6	Neg	Neg	I + RM	23.5	Moderate	V^a^	Resolved	1.0	17	Neg	Neg	+76.8%
7	M/52	31.5	**16.3**	N/A					I + RM + D	26.0	Moderate	V^b^	Resolved	2.1	20	Neg	Neg	+12.0%

**Notes.**

Abbreviations No. patient number M male F female TSH thyroid-stimulating hormone fT4 free thyroxine fT3 free triiodothyronine N/A not available Pos positive Neg negative Rx treatment regimen I interferon RB ribavirin RM remdesivir D dexamethasone TV thyroid volume E heterogenous parenchymal echogenicity M diffuse micronodular parenchymal change V abnormal vascularity

Values in bold represents values out of reference ranges.

Units: age in years; BMI in kg/m^2^; CRP in ng/mL; TSH in mIU/L; fT4 and fT3 in pmol/L.

a Increased vascularity.

b Reduced vascularity.

## Discussion

To our knowledge, this is the first study on the evolution of thyroid volume and thyroiditis features on USG among COVID-19 survivors in the first year post-COVID-19, elucidating the dynamic impact of COVID-19 on the thyroid from a structural perspective. We showed a significant increase in thyroid volume from 2.4 months to 8.7 months after acute COVID-19, where higher SARS-CoV-2 viral load in acute COVID-19 was the only independent predictor of significant increases in thyroid volume from USG1 to USG2. Furthermore, most thyroiditis features on USG1 resolved on USG2. Together with evidence of biochemical recovery from NTIS in the convalescent phase, our findings suggested a transient effect of SARS-CoV-2 on the thyroid gland volume and parenchyma which resolved over 6 months in the later phase of convalescence. Persistent significant thyroid architectural distortion appears unlikely, especially in those without biochemical evidence of thyroiditis in acute COVID-19. Our results could support the evidence-based recommendation that routine surveillance of thyroid USG and thyroid function testing among COVID-19 survivors would not be necessary.

Our previous study evaluated a range of parameters in acute COVID-19 in association with the thyroid volume (Lui et al., 2021a). Among them, only SARS-CoV-2 viral loads in acute COVID-19 showed an independent correlation with the thyroid volume, suggesting a possible direct viral effect of SARS-CoV-2 on the thyroid causing atrophy. The expression of ACE2, the receptor of SARS-CoV-2 entry, in the thyroid provides a plausible mechanistic link explaining the correlation between higher SARS-CoV-2 viral loads and smaller thyroid volumes (Murugan & Alzahrani, 2021a; Rotondi et al., 2021). Autopsy of deceased COVID-19 patients found SARS-CoV-2 nucleocapsid antigen in the epithelial cells lining thyroid follicles in patients (Poma et al., 2021b). Immune genes associated with apoptosis were also found to be upregulated in SARS-CoV-2 positive thyroid glands (Poma et al., 2021a). All these suggested plausible pathways for direct thyroid damage, causing collapse and distortion of the thyroid follicular micro-structure extrapolating from changes reported with SARS-CoV-1 (Wei et al., 2007) and leading to initial thyroid shrinkage at the early phase after COVID-19. Our initial cross-sectional study of thyroid USG features in COVID-19 survivors raised concern of the potential long-term thyroid sequelae of SARS-CoV-2 (Lui et al., 2021a).

**Figure 4:**
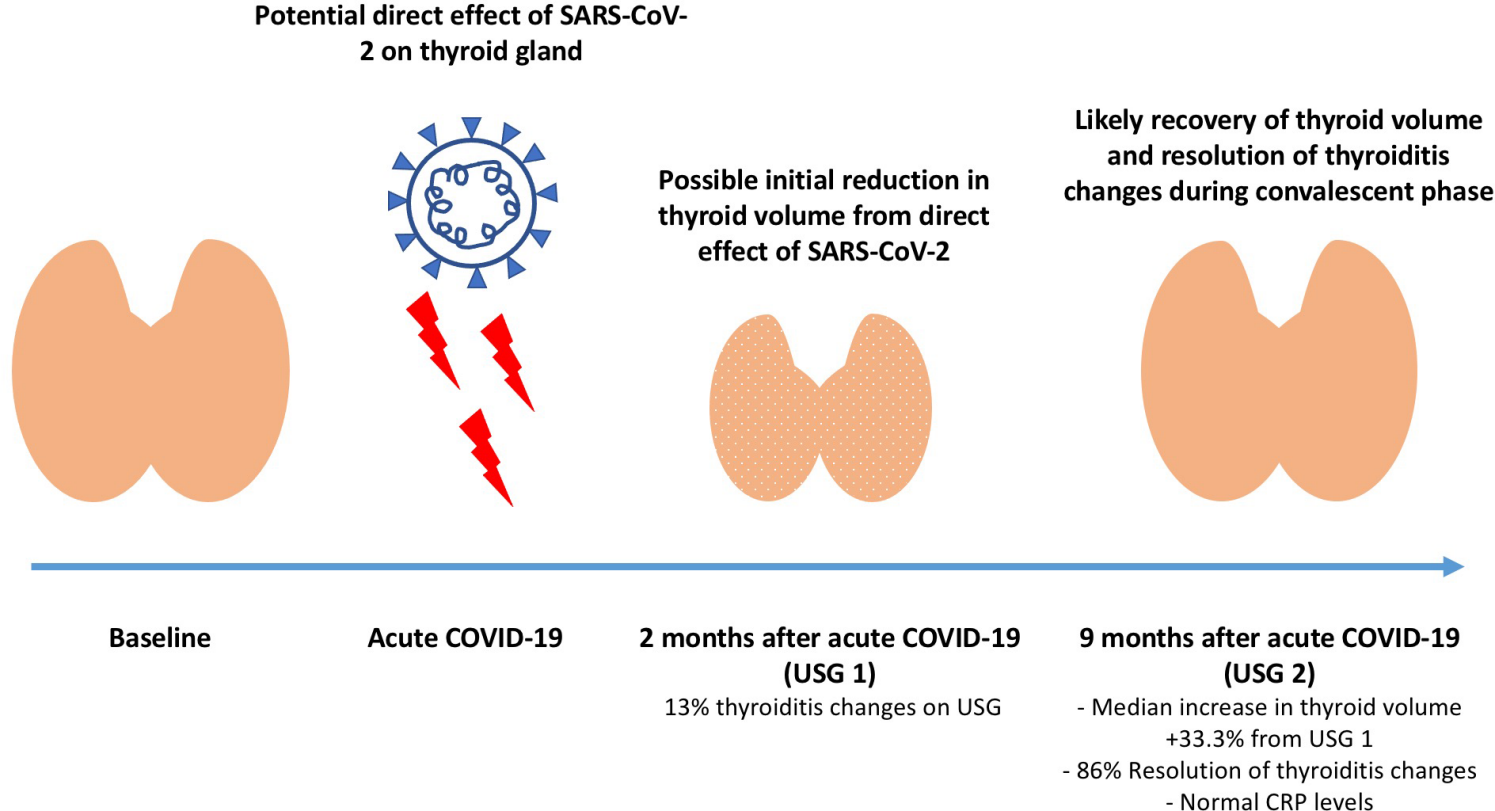
Proposed direct viral effect of SARS-CoV-2 virus on the thyroid gland along the course of COVID-19 infection and recovery. Schematic diagram on the proposed direct viral effect of SARS-CoV-2 virus on the thyroid gland along the course of COVID-19 infection and recovery.

The most significant finding from our current follow-up study was an increase in thyroid volume from USG1 to USG2. We have two hypotheses for this phenomenon: (1) recovery of the thyroid from the initial viral damage to its original state and, therefore, a return to normal volume, or (2) persistent inflammatory cell infiltration *i.e.,* the gland became enlarged. We do acknowledge the limitation of a lack of baseline thyroid volume measurements at the very beginning of the COVID-19 illness in view that all patients were treated in an isolation facility. Nonetheless, inference could be made from the fact that CRP levels were all normal at USG2 and that CRP levels did not correlate with significant increase in thyroid volume, speaking against the latter hypothesis. Previous studies further supported the former hypothesis. Following our first study (Lui et al., 2021a), Urhan et al. (2022) also performed an USG study using a similar study design, of 64 adult COVID-19 survivors receiving thyroid USG at a median of 5.7 months (range: 2–7 months), corresponding to the time between USG1 and USG2. Their study revealed a smaller thyroid volume among COVID-19 survivors compared to healthy controls. Results from the studies by Urhan et al. (2022) and our group could imply the potential decrease in thyroid volume in the early convalescence phase of around 6 months after acute COVID-19. These results could also support our current findings that the thyroid volume increase at USG2, the later convalescent phase, could represent the recovery of the previous thyroid atrophic effect from acute COVID-19 (Fig. 4). COVID-19 comprises an early viral replication phase, and in minority of COVID-19 patients, followed by an inflammatory phase (Ngo et al., 2021). Among the various factors in acute COVID-19 including the inflammatory indices and SARS-CoV-2 viral load, our results summarized in Table 3 showed that SARS-CoV-2 viral load in acute COVID-19 appeared to be a more important determinant of increase in thyroid volume from USG1 to USG2, rather than the degree of inflammation, consistent with the above discussion about the potential direct viral atrophic effect on the thyroid. Moreover, parallel to the thyroid volume increase, we observed an increase in TSH and fT3 levels from acute COVID-19 to USG1, likely representing a recovery from NTIS due to acute COVID-19 (Khoo et al., 2021). Indeed, our group has recently demonstrated that most abnormal thyroid function resolved at 6 months after acute COVID-19 and incident abnormal thyroid function or thyroid autoimmunity was not evident at 6 months after COVID-19 (Lui et al., 2023). The consistent recovery of biochemical and radiological parameters suggested no signal of long-term thyroid sequelae from SARS-CoV-2 infection.

Another important aspect of thyroid USG assessment among COVID-19 survivors was the subclinical ultrasonographic thyroiditis features, as these abnormalities could predict subsequent thyroid dysfunction and would carry implications on the need for thyroid USG surveillance and thyroid function testing among COVID-19 survivors (Rago et al., 2001). None of the patients in our current follow-up study had overt biochemical evidence of thyroiditis in acute COVID-19, in contrast to the study by Pizzocaro et al. (2021) focusing on COVID-19 survivors with SARS-CoV-2-related thyrotoxicosis during acute COVID-19, who would require follow-up for the biochemical abnormalities anyway. Thus, our current follow-up study addressed whether long-term thyroid USG or thyroid function surveillance is necessary for COVID-19 survivors who did not have overt biochemical evidence of thyroiditis during acute COVID-19. Our previous cross-sectional study reported a 13% rate of thyroiditis features (Lui et al., 2021a). Most of these features resolved on USG2, with only one patient having persistent thyroiditis features. This patient had positive anti-Tg noted already in acute COVID-19, suggesting that pre-existing thyroid autoimmunity might account for the thyroiditis features instead of SARS-CoV-2. She also had a lower fT3 level during acute COVID-19, which may suggest a more severe COVID-19 illness and therefore a longer recovery for the thyroiditis features needed. Importantly, we did not observe incident thyroiditis features, thyroid dysfunction or CRP elevation at USG2. Taken together, our serial evaluation of thyroiditis features did not suggest benefits of routine thyroid USG assessment among COVID-19 survivors who did not have overt thyroid dysfunction in acute COVID-19.

Accumulating evidence has refined the definition and the scope of long COVID (Davis et al., 2023). There have been studies showing the increased risk of cardiovascular diseases after acute COVID-19, including venous thromboembolism (Bularga, Newby & Chapman, 2023) and both non-ischaemic and ischaemic heart diseases (Xie et al., 2022). Postulated mechanisms included endothelial dysfunction and prothrombotic effects. On the other hand, together with our current study, evidence has suggested that thyroid gland is not a major target organ in the context of long COVID, from both biochemical and structural perspectives.

Our study had the strength of a prospective study with a well-defined protocol and systematic assessments of thyroid USG, thyroid function and autoantibodies, and inflammatory markers. Nonetheless, there were some limitations. Firstly, the sample size was rather small. Secondly, baseline USG was not available, as all patients were treated in an isolation facility in acute COVID-19 based on the local infection control policy. Thirdly, although inter-observer variability was eliminated by having the same operator performing USG, intra-observer variability inevitably existed. To minimize the effect of intra-observer variability, we believed a cut off of ±15% based on existing literature (Schlögl et al., 2006; Andermann et al., 2007) could readily eliminate minor changes due to measurement errors. Fourthly, we did not have a control group with no exposure to SARS-CoV-2. Fifthly, our study consisted of mostly non-severe COVID-19 patients, so our results might not be generalized to survivors from the most severe form of COVID-19. Last but not least, whether the dynamic changes in thyroid volume after COVID-19 are exclusive to SARS-CoV-2, or represent recovery from acute illnesses or viral infections in general remains to be elucidated, as we did not have a non-COVID-19-illness cohort for comparison. Yet to our knowledge, longitudinal data on thyroid USG findings after common viral infections such as influenza were not available in the literature.

## Conclusion

We observed an increase in thyroid volume from 2 months (USG1, the early convalescent phase) to 9 months (USG2, the later convalescent phase) after COVID-19, where higher SARS-CoV-2 loads in acute COVID-19 independently correlated with the significant increase in thyroid volume. The thyroiditis features on USG observed in the early convalescent phase largely resolved in the later convalescent phase. Together with evidence of biochemical recovery from NTIS in the convalescence, our findings suggested a transient atrophic effect of SARS-CoV-2 on the thyroid gland volume and parenchyma which resolved in the later convalescent phase. Our study provided important reassurance that permanent thyroid destruction was unlikely among COVID-19 survivors without clinically overt thyrotoxicosis during acute COVID-19.

##  Supplemental Information

10.7717/peerj.15034/supp-1 Data S1 Raw data

10.7717/peerj.15034/supp-2 Table S1 Comparison between patients with and without significant thyroid volume increase (excluding one patient who had thyroid volume decrease)

10.7717/peerj.15034/supp-3 Table S2 Multivariable logistic regression analysis of the factors in COVID-19 associated with subsequent significant increase in thyroid volume, excluding one patient with significant thyroid volume decrease

## Additional Information and Declarations

### Data Availability

The following information was supplied regarding data availability: The data is available at Mendeley: Lui, David Tak Wai; Fung, Man Him Matrix (2022), “A prospective follow-up of thyroid volume and thyroiditis features on ultrasonography among survivors of predominantly mild to moderate COVID-19”, Mendeley Data, V1, doi: 10.17632/38z47mt7vr.1.
