# Supplementary material for: A prospective follow-up of thyroid volume and thyroiditis features on ultrasonography among survivors of predominantly mild to moderate COVID-19
Source: PeerJ. 2023 Mar 17;11:e15034. doi: 10.7717/peerj.15034 (PMC10026714; doi:10.7717/peerj.15034)
Supplement: Table S1 [file peerj-11-15034-s002.docx]

**Supplementary Table 1. Comparison between patients with and without significant thyroid volume increase (excluding one patient who had thyroid volume decrease)**

|  | **Significant increase in thyroid volume** | **No significant increase in thyroid volume** | **P value** |
| --- | --- | --- | --- |
| Number of patients | 32 | 21 | -- |
| Age (year) | 48.7±16.1 | 47.7±12.8 | 0.942 |
| Male | 20 (62.5%) | 13 (61.9%) | 0.965 |
| BMI (kg/m^2^)^a^ | 25.7 (22.6 – 29.1) | 27.0 (23.5 – 30.1) | 0.452 |
| Charlson Comorbidity index ≥1 | 5 (15.6%) | 3 (14.3%) | >0.999 |
| Hypertension | 5 (15.6%) | 5 (23.8%) | 0.456 |
| Diabetes mellitus | 1 (3.1%) | 1 (4.8%) | >0.999 |
| Smoking | 5 (15.6%) | 0 (0%) | 0.144 |
| Drinking | 6 (18.8%) | 3 (14.3%) | >0.999 |
| **Parameters in acute COVID-19** | | | |
| SARS-CoV-2 PCR Ct value^a^ | 19.9 (15.6 – 28.3) | 26.8 (21.0 – 29.1) | **0.006** |
| CRP (mg/dL)^a^ | 1.02 (0.32 – 2.82) | 2.02 (0.53 – 7.50) | **0.017** |
| TSH (mIU/L)^a^ | 0.87 (0.73 – 1.50)^b^ | 0.93 (0.57 – 1.45)^c^ | 0.744 |
| fT4 (pmol/L) | 17.0 (15.0 – 18.5)^b^ | 17.0 (15.0 – 19.0)^c^ | 0.831 |
| fT3 (pmol/L) | 3.8 (3.4 – 4.4)^b^ | 4.1 (3.8 – 4.3)^c^ | 0.287 |
| Anti-TPO positivity | 3/21 (14.3%) | 1/12 (8.3%) | >0.999 |
| Anti-Tg positivity | 3/21 (14.3%) | 0/12 (0%) | 0.284 |
| **Baseline COVID-19 severity** | | | 0.157 |
| Mild | 22 (68.8%) | 10 (47.6%) |  |
| Non-mild | 10 (31.2%) | 11 (52.4%) |  |
| **COVID-19 treatments** | | | |
| Remdesivir | 11 (34.4%) | 10 (47.6%) | 0.335 |
| Interferon beta-1b | 11 (34.4%) | 9 (42.9%) | 0.533 |
| Dexamethasone | 2 (6.3%) | 7 (33.3%) | **0.021** |
| Ribavirin | 3 (9.4%) | 4 (19.0%) | 0.415 |
| Clofazimine | 2 (6.3%) | 2 (9.5%) | >0.999 |
| Tocilizumab | 0 (0%) | 1 (4.8%) | 0.396 |

^a^logarithmically transformed before analysis; ^b^n=21; ^c^n=13

Abbreviations: BMI, body mass index; CRP, C-reactive protein

Note: continuous variables compared with t-test; categorical variables compared with Chi-square test or Fisher’s exact test as appropriate
