# Supplementary material for: A prospective follow-up of thyroid volume and thyroiditis features on ultrasonography among survivors of predominantly mild to moderate COVID-19
Source: PeerJ. 2023 Mar 17;11:e15034. doi: 10.7717/peerj.15034 (PMC10026714; doi:10.7717/peerj.15034)
Supplement: Table S2 [file peerj-11-15034-s003.docx]

**Supplementary Table 2.** Multivariable logistic regression analysis of the factors in COVID-19 associated with subsequent significant increase in thyroid volume, excluding one patient with significant thyroid volume decrease

|  | **Adjusted odds ratio (95% CI)** | **P value** |
| --- | --- | --- |
| SARS-CoV-2 viral load in acute COVID-19^a,b^ | 16.749 (1.431 – 195.976) | **0.025** |
| Baseline CRP in acute COVID-19 (mg/dL)^a^ | 0.769 (0.417 – 1.419) | 0.401 |
| Dexamethasone treatment in acute COVID-19 | 0.208 (0.026 – 1.694) | 0.142 |

Note: p=0.405 in Hosmer and Lemeshow test

^a^logarithmically transformed before analysis

^b^SARS-CoV-2 viral loads were presented as $\frac{1}{SARS-CoV-2 PCR Ct value}$

Abbreviation: CRP, C-reactive protein
